# Detection of the Inflammation Biomarker C-Reactive Protein in Serum Samples: Towards an Optimal Biosensor Formula

**DOI:** 10.3390/bios4040340

**Published:** 2014-10-03

**Authors:** Wellington M. Fakanya, Ibtisam E. Tothill

**Affiliations:** 1 Centre of Biomedical Engineering, Cranfield University, Cranfield, Bedfordshire, MK43 0AL, UK; E-Mail: wellington.fakanya@atlasgenetics.com; 2 Atlas, Derby Court, Epsom Square, White Horse Business Park, Trowbridge, BA14 0XG, UK

**Keywords:** electrochemical immunosensor, C-reactive protein, CRP, biomarker, serum

## Abstract

The development of an electrochemical immunosensor for the biomarker, C-reactive protein (CRP), is reported in this work. CRP has been used to assess inflammation and is also used in a multi-biomarker system as a predictive biomarker for cardiovascular disease risk. A gold-based working electrode sensor was developed, and the types of electrode printing inks and ink curing techniques were then optimized. The electrodes with the best performance parameters were then employed for the construction of an immunosensor for CRP by immobilizing anti-human CRP antibody on the working electrode surface. A sandwich enzyme-linked immunosorbent assay (ELISA) was then constructed after sample addition by using anti-human CRP antibody labelled with horseradish peroxidase (HRP). The signal was generated by the addition of a mediator/substrate system comprised of 3,3,5*'*,5*'*-Tetramethylbenzidine dihydrochloride (TMB) and hydrogen peroxide (H_2_O_2_). Measurements were conducted using chronoamperometry at −200 mV against an integrated Ag/AgCl reference electrode. A CRP limit of detection (LOD) of 2.2 ng·mL^−1^ was achieved in spiked serum samples, and performance agreement was obtained with reference to a commercial ELISA kit. The developed CRP immunosensor was able to detect a diagnostically relevant range of the biomarker in serum without the need for signal amplification using nanoparticles, paving the way for future development on a cardiac panel electrochemical point-of-care diagnostic device.

## 1. Introduction

C-reactive protein (CRP) was first discovered by Tillett and Francis in 1930 [1], and it was the first acute-phase protein to be described. CRP is a sensitive systemic marker of inflammation and tissue damage [2,3]. Although it is considered a non-specific biomarker, its level rises steeply (1000-fold over base line) as a result of tissue damage caused by infections and other acute inflammatory events, including autoimmune diseases and malignancy. Hence, monitoring is considered very useful for screening and also disease management [4]. CRP has evolved from being a postulated marker that can possibly predict cardiovascular events and mortality to a proven direct participant in the pathogenesis of atherosclerotic cardiovascular disease [5]. Research and developments in this area have proved that CRP is instrumental in the initiation of several pathogenic pathways that can cause atherosclerosis, a precursor to cardiovascular disease [6,7,8]. Although, there are still some researchers that are not convinced of its pathogenic role [9]. CRP has been used as a general marker for inflammation and, more recently, for predicting cardiovascular diseases [10,11]. Commercially available methods for CRP analysis using immuno-turbidimetric or immuno-nephelometric methods have been reported with sensitivity of about 3–8 µg·mL^−1^ [12]. Automated clinical instruments using the same techniques have been reported to be able to achieve 0.5–10 µg·mL^−1^ CRP detection [13]. More rapid tests have also been developed, some with relatively low sensitivity (5 mg·L^−1^) [14]. However, these methods are either time consuming or lab-based systems that cannot be easily transferred to point-of-care platforms, which cut the turnaround time of sample to results. In managing cardiovascular disease (CVD) patients, every second counts, as there is a limited window of opportunity to treat the patient. With stretched resources in most public health organizations, there is a need to quickly discriminate and prioritize life-threatening conditions and other manageable conditions though rapid point-of-care diagnostic solutions.

The World Health Organization (WHO) lists CVD as the premier cause of death globally, accounting for almost 18 million deaths annually [15]. According to the British Heart Foundation, CVD is the main cause of death in the UK and accounts for almost 198,000 deaths each year, which equates to slightly more than every one in three deaths. As such, the socioeconomic impact of cardiovascular disease is vast, and CVD is attributed to costing the healthcare system in the UK. £14.4 billion annually [16]. The standard 12-lead electrocardiogram (ECG) is currently the single best test to identify patients with acute CVD in most emergency departments [17]. However, it has relatively low sensitivity for the detection of other cardiac condition, like acute myocardial infarction (AMI). The sensitivity of ST-segment elevation for the detection of AMI is 35–50%, leaving more than half of all AMI patients unidentified [17]. Because of the relatively poor sensitivity of the standard 12-lead electrocardiogram (ECG) to detect patients with CVDs, like acute coronary syndrome (ACS), additional diagnostic techniques are used. These involve the use of a panel of cardiac biomarkers, and in some cases, confirmatory imaging techniques are employed [18]. In most emergency care units following the ECG, biomarkers are the second method used to identify patients with potential cardiovascular diseases followed by imaging [19]. Several biomarkers have been used for detecting cardiovascular disease, and these include myoglobin, creatinine kinase-MB (CK-MB), lactate dehydrogenase, aspartate transaminase, glycogen phosphorylase and the most widely used cardiac troponins [20]. Biomarkers have been proven to work better as a panel, especially when consisting of more than two biomarkers. This is because different biomarkers are released into the blood stream at different pathophysiological stages of CVD and often return back to baseline levels after some time [19,20]. Given its strong associations with CVD, as described earlier, CRP was chosen here as a model marker for the development of an electrochemical immunosensor. CRP has potential to give invaluable prognostic information if included as part of a diagnostic panel for detecting CVD [18,21]. A cut-off level of 2–3 μg·mL^−1^ has been reported in the literature for CRP values associated with risk of coronary events [22]. More risk-specific clinical reference ranges for CRP assay are described as ≤1 μg·mL^−1^ for low risk, 1–3 μg·mL^−1^ for medium risk and ≥3 μg·mL^−1^ for high risk [23]. Electrochemical immunosensors are a strong alternative to the traditional immunoassays, which often involve an optical output [24,25]. They offer realistic opportunities to overcome the limitations of the traditional ELISAs and laboratory-based diagnostics by offering the potential for fast, accurate, reliable and affordable diagnostic solutions that are robust and compact [26,27]. Another advantage is the absence of expensive optical components, which has the ability of making electrochemical detection cost effective. Table 1 shows some of the different biosensing technologies for detecting CRP and their detection limits. 

**Table 1:** Biosensors for C-reactive protein detection.

Biosensor Technology Highlights	CRP Detection Limits	Reference
Surface plasmon resonance imaging (SPRi) biosensor	10 pg·mL^−1^	[28]
RNA aptamers on carbon nanotube’s interdigitated electrodes	1–8 μmol·L^−1^	[29]
MOFSET/BJT hybrid transistor-based biosensor	1 pm·L^−1^	[30]
Square wave A/stripping voltammetry	0.5 to 200 μg·mL^−1^	[31]
Thermally controlled piezoresistive microcantilever	1 µg·mL^−1^	[32]
Electrochemical impedance spectroscopy and cyclic voltammetry on carbon nanorods on electrodes	0.001 µg·mL^−1^	[33]
Electrochemical Impedance biosensor	0.04 to 5.84 μg·mL^−1^	[34]
Amperometric biosensor	0.3 to 100 μg·mL^−1^	[35]
Vertical flow immunoassay using flow through films (FTH) and gold nanoparticles	0.01–10 μg·mL^−11^	[36]

Immunosensors can be easily incorporated onto microfluidic platforms, thus cutting the amount of reagents used compared to conventional methods. Electrochemical detection is capable of achieving improved sensitivity, as the detection of a nano-scale current is possible [25,26,27,33,34,35,36].

This paper describes the development of an electrochemical immunosensor for the detection of CRP, as one of the markers for multi-biomarker sensor development. The sensor provides an elegant alternative to the traditional immunoassay techniques and uses a gold working electrode, which has been proven to be superior, both for the binding of antibodies and the generation of a response signal [37]. It has since been established that electrochemical immunosensors are sensitive analytical tools capable of obtaining low detection limits with reduced instrumentation costs [38,39]. A sandwich-based immunoassay was developed in this work, where immobilized monoclonal anti-CRP antibody reacts with free human CRP protein to form an immune-complex, which is detected by a secondary horseradish peroxidase conjugated anti-CRP monoclonal antibody. The transduction of the response was conducted using chronoamperometry, where the current generated from the enzyme reaction was detected by the gold sensor. Investigations were carried out to optimize the sensor performance by looking at alternative sensor inks and different methods of curing the inks. The obtained optimum working conditions were then employed to the best sensor in an attempt to measure the concentration of CRP in buffer solution and, ultimately, in serum samples. The final aim of the development of this immunosensor is to incorporate it as part of a panel on a biosensor device. The final multi-array sensor will incorporate other immunosensors for cardiac biomarkers, like cardiac troponins, which are currently under development.

## 2. Experimental Section

### 2.1. Chemicals and Materials

Potassium chloride (KCl), potassium ferricyanide (K_3_Fe(CN)_6_+3H_2_O), TMB (3,3*'*,5,5*'*-tetramethylbenzidine hydrochloride), PBS (phosphate buffered saline), containing 0.137 M sodium chloride, and phosphate buffer, 0.01 M, pH 7.4, in tablet form, citrate phosphate buffer tablets, hydrogen peroxide, sodium hydroxide and sodium chloride, bovine serum albumin (BSA), 11-mercaptoundecanoic acid (11-MUA) and 3,3-dithiodipropionic acid (DTDPA) were purchased from Sigma-Aldrich (Dorset, UK). Micropipettes were purchased from Eppendorf, UK Pipette tips and 1.5-mL tubes were purchased from Fisher Scientific (Loughborough, UK). Anti-human C-reactive protein, capture antibody and mouse monoclonal C6 to C-reactive protein (AB 8272, anti-human C-reactive protein, HRP-conjugated) detection antibody were bought from (Abcam, Cambridge, UK). Other chemical and materials include: skimmed milk protein (KPL Ltd., Gaithersburg, MD, USA); CRP free serum Fitzgerald (Europa bioproducts, Cambridge, UK); and reverse osmosis (RO, Ultrapure) water (18 M·MΩ·cm), obtained from Milli-Q water system (Millipore Corp., Tokyo, Japan). Glassware and volumetric materials were of analytical grade. ELISA tests were first developed using polystyrene plates, MaxiSorp^®^, purchased from Fisher Scientific, Loughborough, UK. An incubator/shaker from Labsystem iEMS^®^ was used for temperature control incubation. A Florostar^®^ plate reader was used, which was set at appropriate wavelengths, BMG, UK. 

### 2.2. Fabrication of Screen-Printed Gold Electrodes

The electrodes used in this project were designed by Cranfield University and fabricated using the facilities at DuPont Microcircuit Materials (Bristol, UK). Screen-printed gold electrodes (SPGE) consisting of a gold working electrode, a complementary carbon counter electrode and an Ag/AgCl reference electrode, were fabricated using a procedure similar to that described in detail by Noh and Tothill [40]. The substrate material used is PET 125 μm Autotype HT5. All printing inks used were from DuPont Microcircuit Materials (Bristol, UK). The carbon (7102 or BQ221), 7-μm dried thickness, was used for both tracking and the counter electrode, while the working electrode was made from a gold colloid ink (BQ331 variant) printed to make an 18 μm-thick layer with a width of 5 mm in diameter, giving a 19.6-mm^2^ planar area, which was printed on a graphite ink layer of similar dimensions. The reference electrode was comprised of Ag/AgCl (5870 or 5880) with an approximate dried thickness of 18 μm. Finally, the electrodes were encapsulated in a polymer coating (5036 blue variant) that had an approximate dried thickness of 4 μm and, unless stated, cured by oven drying at 120 °C for 30 min. The electrodes were then tested for quality assurance using a multi-meter before use.

An Autolab Electrochemical Analyzer with general-purpose electrochemical software, GPES 4.9, was used for electroanalysis (Metrohm, Ultrech, Netherlands). The electrodes were connected to an edge connector and then connected to the electrochemical analyser using a modified connector (Maplin, UK).

### 2.3. Optimizing the CRP Assay on the Gold Electrode

The optimization of the gold electrode was adapted from a procedure described by Salam and Tothill [24]. Antibody immobilization on the gold working electrode using passive adsorption was first conducted to find the best concentrations for both the capture and detection antibody. The electrodes were re-baked at 120 °C for 30 min, first to remove any oxidized materials, and washed with distilled water before blow drying with a nitrogen gun. For the coating antibody investigations, the working electrode was coated with 10 µL of mouse anti-CRP monoclonal antibody dissolved in 0.05 M carbonate-bicarbonate buffer, pH 9.6. Different concentrations of the coating antibody (5, 10, 20, 50, 100 µg·mL^−1^) were deposited in triplicates and allowed to incubate overnight at 4 °C. The electrode surface was then gently washed with phosphate buffer saline with 0.05% Tween 20 (PBS-T) two times and rinsed with distilled water before air-drying with nitrogen. The working electrode surface was blocked for 30 min at 37 °C with 50 µL skimmed milk protein, 1:10 dilution in PBS, in a controlled humid environment. The electrodes were then washed in a similar manner as described above, and a standard concentration of CRP (20 µL 10 µg·mL^−1^) was added to all working electrodes before being incubated for 2 h at 37 °C.

The electrode was then washed, and a conjugated rabbit monoclonal antibody with HRP (5 µg·mL^−1^) with 1:40 dilution milk protein was added and incubated for 30 min at 37 °C. The electrodes were then washed and connected to an edge connector, which is connected with a multi-channel AUTOLAB electrochemical instrument (Ecochemie, Utrecht, The Netherlands). All measurements were performed by adding 100 µL of 4 mM TMB and 0.06% H_2_O_2_ in 0.05 M citrate phosphate buffer plus 0.1 M KCl with chronoamperometry methods at a −200 mV constant current and run for 200 s using electrochemical GPES 4.7 software. 

Similarly, the same was conducted for investigating the optimized conditions for the detection antibody. The coating antibody was standardized at the optimum concentration (60 µg·mL^−1^), and the antigen was also fixed at 1 µg·mL^−1^, but several detection antibody concentrations were used (10, 20, 30, 50, 100 µg·mL^−1^) to investigate the optimum concentration. Other conditions that were optimized were incubation times and temperatures.

### 2.4. Cyclic Voltammetry

Cyclic voltammetry was used to evaluate the electrochemical behaviour of the electrodes, to characterize the surface of the gold working electrode and to determine its performance. This was done by measuring the electrochemical signal from the redox reaction potassium ferro-/ferri-cyanide at varying scan rates (10 mV/s, 20 mV/s, 40 mV/s, 60 mV/s, 80 mV/s). The controlled reaction in a supporting electrolyte solution of 0.1 M KCl on a pre-treated screen-printed gold electrode results in a cyclic voltammetric (I-E) curve for the oxidation of ferrocyanide (1 mM) to ferricyanide. The analysis of the electrodes using cyclic voltammetry is based on the fact that a cyclic voltammogram of a fully-reversible system will display a known set of characteristics governed by a series of equations [41]. One of the major equations is the Randles–Sevcik equation, which at room temperature is:

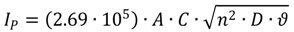

where n is the number of electrons, *A* the electrode area (in cm^2^), *C* the concentration (in mol/cm^3^), *D* the diffusion coefficient (in cm^2^/s) and ʋ the scan rate (in V/s). The equation can be used to estimate the percentage of the active surface of the working electrode [42]. The relationship between the oxidation and reduction peaks to the square root of the scan rate was also used in the analysis. This relationship is important in showing the effectiveness of the diffusion of the electrolytes on the sensor surface. It is a good indicator for the quality of the electrode, particularly when unstirred, highly electrolytic solutions are used.

### 2.5. Investigating Different Ink Compositions and Curing Techniques

Cyclic voltammetry and immunoassays were carried out on three sets of electrodes with different ink compositions. The optimized CRP assay was used on each of the three electrodes tested, given the pseudonyms JA, JC and JD, printed at DuPont. The composition of the electrodes were based on the DuPont printing paste codes and include: JA (carbon ink 7102/gold ink BQ331/Ag/AgCl 5870/encapsulating 5036 blue), JC (carbon ink 7102/gold ink BQ331/5870/Ag/AgCl 5880/encapsulating 5036 blue) and JD (carbon ink BQ221/gold ink BQ331/Ag/AgCl 5880/encapsulating 5036 blue). Three different concentrations of CRP (from human serum) were used in triplicates for each of the sensors. The electrodes were then washed and connected to a multi-channel AUTOLAB. All measurements were performed by adding 100 µL of 4 mM TMB and 0.06% H_2_O_2_ in 0.05 M citrate phosphate buffer plus 0.1 M KCl with chronoamperometry methods at a −200 mV constant current and run for 200 s using electrochemical GPES 4.7 software. The cyclic voltammetry method described earlier was carried out on each set of electrodes to find the relationship of oxidation and the reduction peaks compared to the scan rates. Two different sensor curing methods for setting the ink on the substrate after screen printing were also investigated using the same method described above. The first involved curing the electrodes in a static/box oven with a set temperature of 130 °C for 20 min, while the second involved drying the sensors for 4 min in a conveyor belt drier that utilised both infrared (IR) and convection heat. 

### 2.6. Investigating Different Immobilization Techniques

Two covalent immobilization techniques were investigated using techniques similar to that described by Salam and Tothill [24]. The gold working electrode was plasma cleaned in oxygen-free nitrogen at 50 w for 2.5 min, washed in ethanol and immersed overnight at room temperature in respective solutions of 3% w/v 3,3-dithiodipropionic acid (DTDPA) and 3% 11-MUA in ethanol at the same conditions. The electrodes were then washed with deionized water and dried using a gentle N_2_ flow. Twenty microliters of an equal volume of Carbodiimides (EDC)–N-hydroxysuccinimide (NHS) (0.4 M EDC and 0.1 M NHS prepared in distilled water) was applied to activate the electrode surface for 15 min at room temperature. The electrodes were then washed and dried using a N_2_ flow, and 10 µL of mouse monoclonal anti-CRP solution, 50 µg·mL^−1^ dissolved in 0.05 M carbonate bicarbonate buffer, pH 9.6, were deposited over the surface for immobilization. Exposed ester groups were blocked with 20 µL of methanolamine-HCl. The electrodes were then washed as described above, and 20 µL of standard concentrations of CRP (0, 25, 50, 100 ng·mL^−1^) were added to the working electrodes and incubated for 1 h at 37 °C. The electrodes were then washed, and a HRP conjugated mouse monoclonal antibody against human CRP (5 µg·mL^−1^) in 1:40 dilution milk protein was added to the electrode and incubated for 30 min at 37 °C, washed and used for the assay. 

### 2.7. Comparing the SPGE with the ELISA

The developed CRP assay on the screen-printed gold electrodes was compared to the human CRP immunoperoxidase assay, which was purchased from Immunology Consultants Laboratory (Newburg, NY, USA). The three techniques were used to detect the amount of CRP antigen spiked in commercial serum. All serum samples were diluted 200 times with the sample diluent buffer. Six samples with unknown concentrations of CRP were prepared by spiking a random amount of antigen into the commercially acquired serum. The spiked serum samples were then analysed in triplicates using the optimized ELISA prepared in this work, the commercial ELISA, as well as the optimized immunosensor. Standard curves were plotted, and the unknown concentrations of CRP in the samples were interpolated and the results compared. The limit of detection of all of the methods was established. Standard curves were plotted using relative intensities normalized against the highest output at concentration, 100 ng·mL^−1^, of the antigen. The unknowns were interpolated, and the limit of detection of both methods was established.

### 2.8. Calibration and Interpretation of the Data

ELISA and immunosensor calibration curves were fitted by non-linear regression using the following four parameter logistic function [43].


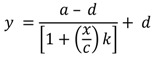


In this equation, *y* is the response or current obtained, *a* and *d* are the maximum and minimum values of calibration curve, respectively, *x* is the concentration at the EC_50_ value, the CRP concentration, and *k* is the slope. In this work the limit of detection (LOD) was defined as the concentration of CRP that is equivalent to three-times the value of the standard deviation (σ). This was calculated based on the following equation, where σ becomes the standard deviation of the zero value [43].


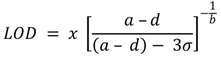


In this equation, *a* = the minimum asymptote of the estimated response at zero concentration, *d* = the maximum asymptote of the estimated response at infinity concentration, *x* represents the concentration of the analyte and *b* is the slope of the curve. 

## 3. Results and Discussion

### 3.1. Optimization of the ELISA Micro-Plate-Based Assay

First, the CRP assay was developed using a microtiter plate in order to ensure that both capture and detection antibodies are sensitive towards CRP. A checkerboard concentrations method was used to optimize and select both the coating and the detection antibodies. A final protocol of 100 µL, 6 µg·mL^−1^, of mouse monoclonal anti-human CRP capture antibody was used for coating, and 100 µL of 0.1 µg·mL^−1^ HRP conjugated rabbit monoclonal anti-human CRP antibody were added as the detection antibody. Different concentrations of the CRP 100 µL (0, 0.5, 1, 5, 10, 20, 40, 60, 80, 100 ng·mL^−1^) were used to produce the standard curve for the assay. The standard curve for the optimized ELISA assay for CRP gave a detection limit of 1.9 ng·mL^−1^, which is comparable to those available on the market. 

### 3.2. Investigating Different Ink Drying Regime

**Figure 1:**
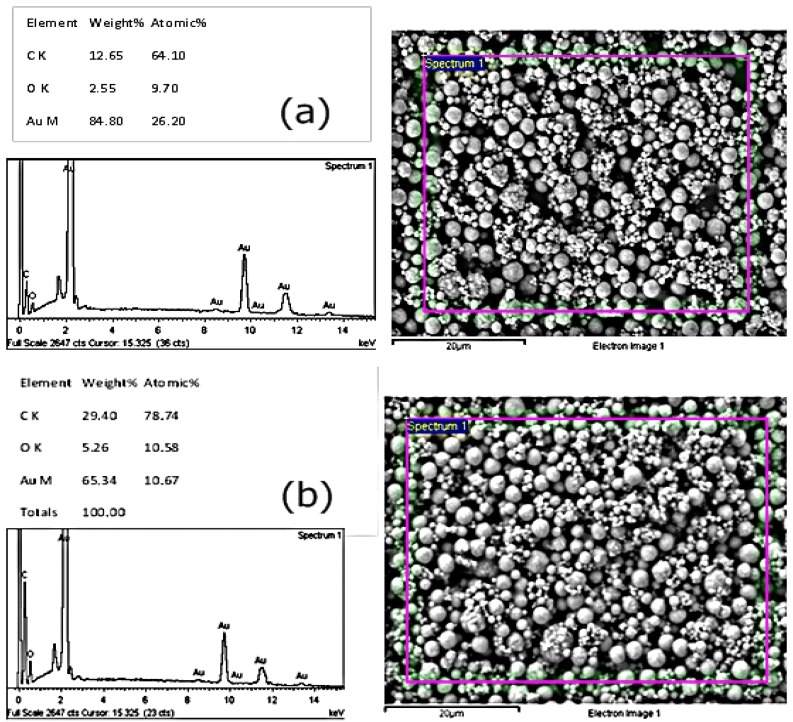
The scanning electron microscope analysis of differently-cured gold inks: (**a**) alternatively-dried sensors and (**b**) infrared-dried sensors.

Two methods of curing the electrodes were investigated, which include alternative drying (oven at 120 °C for 2 h) and infrared (IR) drying using the JD electrode formulations. Infrared dryers are capable of drying screen-printed inks in 15 min, and therefore, are beneficial to shortening the fabrication time of the screen-printed electrodes. The method used to dry the inks is therefore important in characterizing the inks and studying their performance. Cyclic voltammetry was used to characterize the electrodes after drying using potassium ferrocyanide. The results indicated that electrodes that were dried using the alternatively drying method gave higher oxidation and reduction peaks, thus making them more sensitive than the IR dried sensors. The sensors also showed better reproducibility and sensitivity results with the immunoassay constructed on the sensor surface when the alternative drying method was used. These findings were affirmed by environmental scanning electron microscopy (ESEM), which showed a composition analysis with higher amounts of exposed gold in the oven-dried sensors (84.8%) when compared to the infrared-dried sensors (65.34%). A complete summary of The ESEM results is shown in Figure 1. Although the IR drying may have commercial advantages on speeding up the production of the sensors, it is evident that this has an impact on the sensor surface composition. The results in this work show that the conventional oven drying of the working electrode results in better performing gold electrodes for the immunosensor performance, and thus, the electrodes used in this work were cured using the conventional drying method for the gold electrode. This can potentially be explained by the fact that IR drying is a rapid, high temperature drying process, which can trigger convection currents on the ink components. This, in turn, forces less dense nonconductive components of the ink to dominate the surface, thereby insulating against free electron transfer and, thus, affecting the surface of the electrode [44,45,46]. This was seen here also for the immunosensor performance. 

### 3.3. Investigating Different Ink Compositions

**Figure 2:**
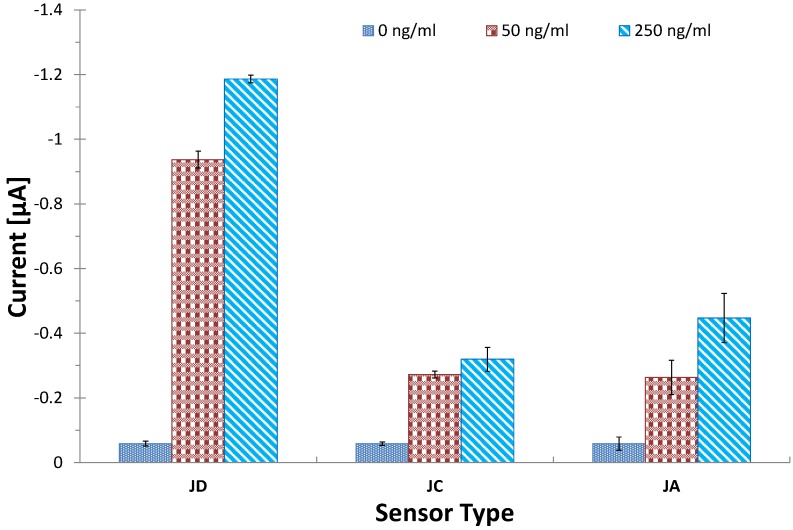
The performance of the JD, JC and JA screen-printed electrodes. Fifteen microliters of 50 µg·mL^−1^ of mouse monoclonal antibody against human CRP in 0.05 M carbonate bicarbonate buffer, pH 9.6, were immobilised on the working electrode by incubation for 1 h at 37 °C. HRP conjugated anti-CRP solutions, the equivalent of 5 µg·mL^−1^ premixed for 30 min with antigen solutions (0, 50, 250 ng·mL^−1^ in 1:40 milk solution in PBS), was added and incubated for 2 h at 37 °C after a washing step. Electrodes were connected to an analyser, and 100 µL of substrate solution were added before amperometric measurements were done at a potential of −200 mV, CV = 11.4%; the bars represent the standard error of the means (n = 4).

The formulations of the inks used in screen-printed electrodes are the proprietary knowledge of the company producing it and remain guarded for commercial reasons. The performance of the inks differs with each formulation and the intended application. In this experiment, three types of electrodes were assembled with different ink combinations, which were then tested for their electrochemical performance using cyclic voltammetry and as an immunosensor platform. The current optimized CRP assay was done on each of the three electrodes, JA, JC and JD. The results obtained are shown in Figure 2, and these indicated a similarity between the performance of the electrodes, JC and JA. This shows that both variants of the Ag/AgCl, namely 5880 and 5870, yielded similar performance on the electrodes. The experiments also showed that JD gave superior performance on both the electrochemical and the immunosensor experiments. This may be because of the different carbon ink used in this sensor (BQ221). Our conclusions are that there was no significant difference in terms of performance between the 5870 and the 5880 Ag/AgCl variants. We also conclude that the BQ221 carbon performs better than the 7102 variant.

### 3.4. Optimization of Coating Antibody on SPGEs

Significantly more concentrated coating antibody solutions were found to be required to give detectable signals on SPGE than on ELISA, especially for the detection of low CRP concentrations. This is due to the sensor surface morphology and the low sample volumes used in the assay, hence the limited surface area for binding on the SPGEs compared to the ELISA. Experiments were conducted to find the optimal capture and detection antibody concentration for the SPGE assay for CRP analysis using the JD electrodes. 

For the coating antibody, the general trend indicated that the best coating antibody concentration was 50 µg·mL^−1^. The results for the detection antibody showed that there was an increase in signal with increasing concentration of the detection antibody. This appears to reach a plateau at a 30 µg·mL^−1^ concentration, where the highest signal was achieved. The drop in the signal after50 µg·mL^−1^ of the coating antibody can be attributed to packing impedance. The response also shows a plateau on further increases in the detection antibody concentration, and this could be due to assay saturation.

### 3.5. Investigating Other Immobilization Methods

**Figure 3:**
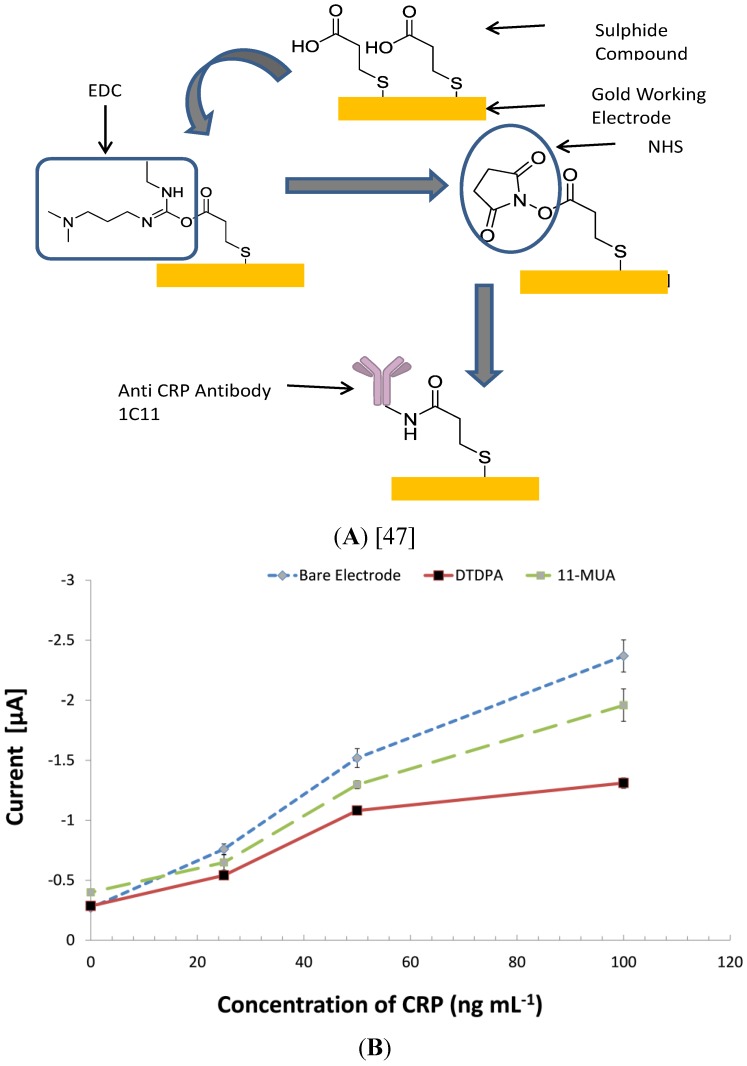
The impact of different covalent surface modifications on the assay performance of C-reactive protein on screen-printed gold electrodes (SPGEs). Two sets of electrodes were chemically treated with self-assembled monolayers (11-mercaptoundecanoic acid (11-MUA) and 3,3-dithiodipropionic acid (DTDPA)) to facilitate covalent coupling of the CRP antibody 1c11 ((**A**) with DTDPA). Optimized electrochemical immunoassays were then conducted to evaluate the effect of self-assembled monolayers (SAM) on the assay performance (**B**). CV = 9.7%; the bars represent the standard error of the means (n = 4).

A standard assay procedure described in Section 2.6 was used to investigate the benefits of covalent coupling using self-assembled monolayers on JD screen-printed gold electrodes, and the results are shown in Figure 3. The results showed that the control signal of 11-MUA coated chips had a higher background signal than the other two immunosensors (Bare and DTDPA). This may have been caused by molecules trapped in the lattice of the 11-MUA monolayer. 

The 11-MUA monolayer has long carbon spacers due to its chemical structure; this means that there is a considerable distance between its reactive groups, and this, in turn, influences the distance between the sensor surface and the ligand. Unlike smaller molecules, like DTDPA, with short carbon spaces, long-chain SAM molecules have been proven to trap proteins non-specifically [48]. In this experiment, there were no obvious advantages of using the covalent coupling technique on the screen-printed gold electrodes. Notable advantages of covalent coupling in immunosensors have been emphasized in the literature, and these include enhancing the stability of the immobilized biomaterials on the sensor surface, as well as prevention of desorption of the immobilized material. Another notable advantage is the ability of monolayers to increase binding capacity, due to controlled orientation; this has been attributed as one of the major pros for using covalent immobilization techniques on sensor surfaces. Controversially, many scientists developed and publicized complex procedures of covalent binding, but they lack quantitative demonstration of the advantages compared to passive adsorption [48,49]. Biosensing surfaces functionalized with alkanethiol SAMs have been reported to suffer from non-specific binding due to non-immobilized bio receptors and imperfectly formed monolayers on the polycrystalline gold grain structure of the sensor surfaces [49]. Several techniques have been tried to control this, especially through de-activating the head group with ethanolamine-HCl [50,51,52]. Blocking with ethanolamine-HCl was conducted in our work, although non-specific binding was still observed. Non-specific binding on the 11-MUA coated chips may also have been caused by imperfect monolayers forming on polycrystalline gold surfaces and the unevenly distributed gold surface. As the performance of passive adsorption was meeting the diagnostic requirements for a CRP assay, no further input on covalent immobilisation was pursued for the development of this immunosensor.

### 3.6. Comparing the CRP Assay on SPGEs against a Commercial ELISA Kit

**Figure 4:**
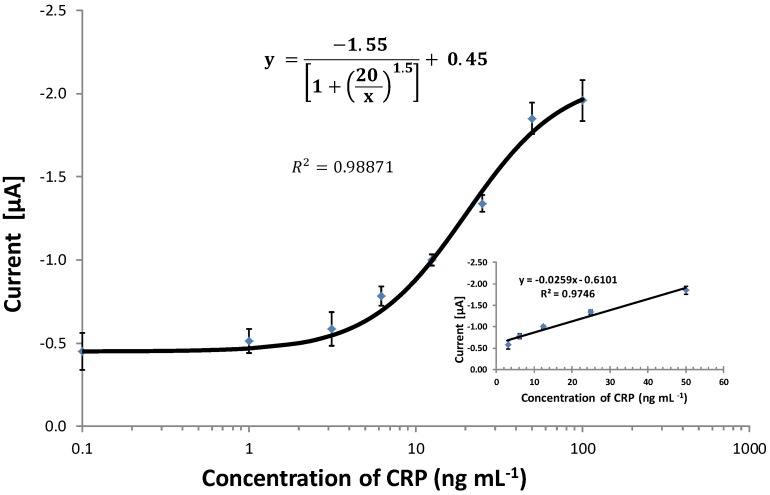
The optimised logarithmic standard curve for the C-reactive protein assay in spiked serum samples on the screen-printed gold electrodes (SPGEs). Electrodes were coated with coating antibody (60 µg·mL^−1^) incubated for 2 h at 37 °C, washed and blocked (10% milk protein). Known concentrations of CRP antigen in serum were premixed with the detection antibody to give appropriate final concentrations. The premixed solution was incubated for 1 h on a rotator. A 20-µL sample was then deposited on the blocked working electrodes and incubated for 1 h. The electrodes were washed, the substrate added and chronoamperometry measurements conducted at a −200 mV potential run for 200 s using Autolab. CV = 9.8%; the bars represent the standard error of the means (n = 4). LOD = 2.6 ng·mL^−1^.

The optimized immunoassay for CRP, using passive adsorption, on the JD gold working electrode was then conducted for a range of biomarker concentrations in spiked serum samples, and the resulting standard curve is shown in Figure 4. The curve fitting was carried out using the four-parameter curve fit described by Warwick [43]. The final developed SPGE immunosensor exhibited a linear range from 2.2 to 100 ng·mL^−1^. This is well in the range for the detection limit required for the standard use of CRP as a biomarker; thus, the immunosensor demonstrated the feasibility of detecting CRP at the required concentration level without the need to further amplification of the signal. 

**Figure 5:**
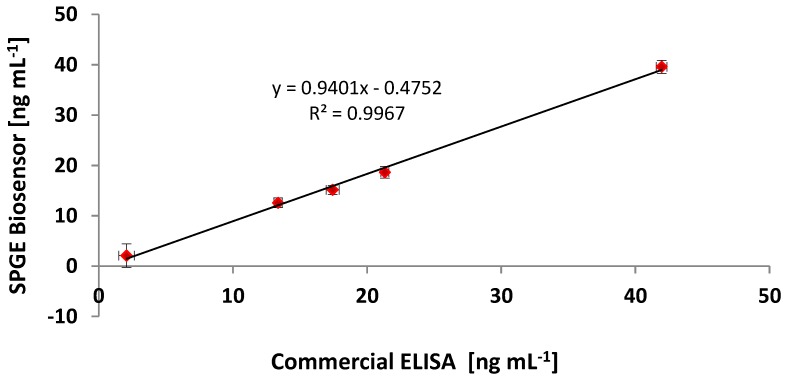
The correlation of the developed immunosensor and the commercial ELISA assay using the same randomly spiked serum samples analysed using both methods.

The developed CRP assay on the screen-printed gold electrodes was then compared to an ELISA assay co-developed in this work using the same reagents. Further comparisons were also done using a commercially acquired ELISA kit (Human CRP immuno peroxidase assay), which was purchased from Immunology Consultants Laboratory, Newburg, USA. The comparison between the three detection methods was done using commercial CRP-free serum samples spiked with CRP. The standard curves were plotted as relative intensities normalized against the highest output at concentration, 100 ng·mL^−1^, of the antigen. Both exhibited the expected sigmoid shape on the logarithmic scale. Figure 5 shows a correlation curve between the immunosensor and the commercial ELISA. The ability of the immunosensor to quantitate unknown CRP levels in randomly spiked serum is also demonstrated in Table 2. The standard curve in Figure 4 shows that the limit of detection for the biosensor was 2.6 ng·mL^−1^ when compared to the commercial ELISA of 1.9 ng·mL^−1^. However, there were similarities in the gradient of the slope, R^2^ 0.99. Direct comparison of the results of these two methods was plotted, indicating a linear relationship, as shown in Figure 5. 

**Table 2:** Analysis of randomly spiked serum samples with different concentrations of CRP using both the commercial ELISA and the developed immunosensor.

Sample	Commercial ELISA		Optimised ELISA *		Immunosensor (SPGEs)	
	(ng·mL^−1^)	CV (%)	(ng·mL^−1^)	CV (%)	(ng·mL^−1^)	CV (%)
Negative Control	1.05	8.57	1.23	13.82	0.98	11.22
Sample 1	21.32	5.48	20.04	6.76	18.64	1.61
Sample 2	17.43	5.21	16.38	6.43	15.13	3.19
Sample 3	13.36	7.24	12.56	8.94	12.57	2.41
Sample 4	2.07	9.48	1.94	11.83	2.09	9.59
Sample 5	41.96	3.03	39.44	3.74	39.58	1.02
Positive Control	77.32	0.53	74.58	0.88	75.36	0.64

* The ELISA assay developed and optimized in this work.

## 4. Conclusions

An electrochemical immunosensor based on a gold working electrode fabricated using screen-printing technology was developed for the detection and analysis of CRP in serum samples. As mentioned earlier, the clinical reference ranges for the CRP assay are ≤1 μg·mL^−1^ (low risk), 1–3 μg·mL^−1^ (medium risk) and ≥3 μg·mL^−1^ (high risk) [23]. The performance of the CRP immunosensor was competent to satisfy these parameters, as the developed electrochemical immunosensor has an LOD of 2.6 ng·mL^−1^ and spans a dynamic range up to 100 ng·mL^−1^. However, when the dilution factor was taken into consideration for the samples, the final dynamic range of the immunosensor was 0.52 µg·mL^−1^ to 20 µg·mL^−1^ in serum. The need to dilute all serum samples with PBS-Tween played a significant role in the attainment of the diagnostic objectives of the assay, mainly for two reasons, which are removing the matrix effect and ensuring that high levels of CRP can be detected easily. Although the effects of dilution may affect assay resolution and lower assay sensitivity, CRP is present in a relatively high concentration in healthy subjects, unlike other CVD biomarkers, like cardiac troponins, which are scarce in healthy individuals. 

Three experiments were conducted to evaluate the effect of different curing conditions on the performance of the electrode. The curing (drying) conditions of an electrode play a major role in the rate and the cost of the production of electrodes [44,45,46]. Different techniques are constantly being investigated to improve the production process by finding faster and cheaper curing alternatives. Infrared drying is a faster and cheaper method than conventional oven drying. The results from this work showed that the conventional drying of the gold working electrode resulted in better performing electrodes. There was a clear difference in both the chemical composition and the morphology of the constituent particles on the gold surface. The ESEM showed that the amount of the exposed gold on the infrared-dried sensors (65.34%) was less than that from the conventionally-dried electrodes (84.8%). This may explain the superior electrochemical behaviour of the conventionally-dried electrode, as shown by the cyclic voltammetry and the immunoassay results. The developed assay proved successful in detecting CRP in serum samples at diagnostically relevant concentrations and can thus be developed further to contribute to a diagnostic panel for diagnosing CVD. 
